# The absolute lymphocyte count accurately estimates CD4 counts in HIV-infected adults with virologic suppression and immune reconstitution

**DOI:** 10.7448/IAS.17.4.19680

**Published:** 2014-11-02

**Authors:** Barnaby Young, David Lye, Oon Tek Ng, Yee Sin Leo

**Affiliations:** Infectious Diseases, Tan Tock Seng Hospital, Singapore

## Abstract

**Introduction:**

The clinical value of monitoring CD4 counts in immune reconstituted, virologically suppressed HIV-infected patients is limited. We investigated if absolute lymphocyte counts (ALC) from an automated blood counting machine could accurately estimate CD4 counts.

**Materials and Methods:**

CD4 counts, ALC and HIV viral load (VL) were extracted from an electronic laboratory database for all patients in HIV care at the Communicable Diseases Centre, Tan Tock Seng Hospital, Singapore (2008–13). Virologic suppression was defined as consecutive HIV VLs <400 days apart, <200 copies/mL. CD4 counts and same day ALCs were collected during virologic suppression from the first CD4> 300 cells/mm^3^. CD4 counts were estimated using the CD4% from the first value >300 and an ALC 181–540 days later.

**Results:**

A total of 1215 periods of virologic suppression were identified from 1183 patients, with 2227 paired CD4-ALCs available for analysis. 98.3% of CD4 estimates were within 50% of the actual value. 83.3% within 25% and 40.5% within 10%. The error pattern was approximately symmetrically distributed around a mean of −6.5%, but significant peaked and with mild positive skew (kurtosis 4.45, skewness 1.07). Causes for these errors were explored. Variability between lymphocyte counts measured by ALC and flow cytometry did not follow an apparent pattern, and contributed to 32% of the total error (median absolute error 5.5%, IQR 2.6–9.3). The CD4% estimate was significantly lower than the actual value (t-test, p<0.0001). The magnitude of this difference was greater for lower values, and above 25%, there was no significant difference. Precision of the CD4 estimate was similar as baseline CD4% increased, however accuracy improved significantly: from a median 16% underestimation to 0% as baseline CD4% increased from 12 to 30. Above a CD4% baseline of 25, estimates of CD4 were within 25% of the actual value 90.2% of the time with a median 2% underestimation. A robust (bisqaure) linear regression model was developed to correct for the rise in CD4% with time, when baseline was 14–24 (coefficients: intercept=3.30, CD4=0.939). This improved accuracy from 82.0% to 85.4%, and median error from 11% underestimation to 1% (p<0.0001). Adding time since baseline CD4% into the model increased complexity without significantly improving accuracy.

**Conclusion:**

In virologically suppressed hosts with CD4 ≥300 cells/mm^3^ and percentage ≥14, CD4 counts can be predicted with 85–90% confidence to within 25% of the actual value using the ALC.

